# Validation of the Patient Empowerment Strategies Questionnaire (PES-Q) in Greek adult patients with chronic insomnia: a pilot study on basic psychometric values

**DOI:** 10.1017/S1463423619000616

**Published:** 2019-09-12

**Authors:** Sofia Tsoli, Michael Galanakis, Anna Koumarianou, Gerasimos Makris, August Kapogiannis, George Chrousos

**Affiliations:** 1 Postgraduate Course on Science of Stress and Health Promotion, School of Medicine, National and Kapodistrian University of Athens (NKUA), Athens, Greece; 2 Second Department of Neurology, “Attikon” University Hospital, National and Kapodistrian University of Athens, Athens, Greece; 3 MBA Program, Hellenic Open University (HOU), Athens, Greece; 4 Hematology-Oncology Unit, Fourth Department of Internal Medicine, Attikon University General Hospital, School of Medicine, National and Kapodistrian University of Athens (NKUA), Athens, Greece; 5 Unit of Developmental and Behavioral Pediatrics, First Department of Pediatrics, Aghia Sophia Children’s Hospital, School of Medicine, National and Kapodistrian University of Athens (NKUA), Athens, Greece

**Keywords:** chronic insomnia, Greek sample, patient empowerment, reliability, validation

## Abstract

**Aim:**

The objectives of this study were to validate the Patient Empowerment Strategies Questionnaire (PES-Q) in a Greek sample and to study its psychometric properties in a sample of patients diagnosed with chronic insomnia.

**Background:**

This is a validation of the PES-Q in Attikon General Hospital, School of Medicine, National and Kapodistrian University of Athens. The questionnaire was administered to 93 subjects aged between 18 and 85 years (mean age ± SD: 54.7 ± 15.2, 28% males).

**Methods:**

The criterion validity of the questionnaire was tested with the use of four specific criteria: the Athens Insomnia Scale, the Pittsburg questionnaire (Pittsburg Sleep Quality Index), the Depression, Anxiety and Stress Scale, and the Self-Esteem Scale.

**Findings:**

According to factor analysis results, the structure of the original scale was confirmed by the presence of one main factor in the Greek sample, explaining 40.1% of the variance of PES-Q queries. The questionnaire showed satisfactory reliability (Cronbach’s *α* = 0.887). The results of the current study suggest that the PES-Q may be used as an accurate psychometric instrument for the purposes of chronic insomnia. Future research should examine the psychometric qualities of the PES-Q Greek version in a larger sample.

## Introduction

According to the World Health Organization, empowerment is defined as ‘practices through which individual patients achieve greater control over their decisions and are capable of affecting their health’ (Guadagnoli and Ward, 1988). Nowadays, progressively more and more public health researchers are engaged in the concept of empowerment, focusing particularly on self-efficacy and decision-making (Miller and Harris, 2012). Empowerment suggests that the individual has increased control over his/her life and health. This may seem particularly difficult within modern, hectic environments that are the source of numerous stressors (eg, work demands, family breeding, transportation, etc.) that threaten the individual. These concepts may also extend to the field of health promotion that strives to empower individuals to make healthy lifestyle choices that will prevent chronic disease and morbidity.

Patient empowerment describes a situation in which individuals are encouraged to take an active role in managing their health and become more involved in making decisions about their life (Anderson and Funnell, 2005; Aujoulat *et al.*, 2007). It is actually a positive cognitive process that helps the patient to recognize and learn to proactively manage his/her illness (Funnell and Anderson, 2003). As a process, it involves (a) the exploration of the patient’s thoughts, (b) the therapeutic options that the therapist may offer by increasing the patient’s knowledge about his/her illness, (c) the educational and cooperative communication according to the treatment plan, (d) the patient’s compliance with the guidelines through the satisfaction that he/she feels from the medical care of the staff, the appropriate interaction, and empathizing by the doctor, and (e) the respect of the patient’s rights to join in the decision-making as far as his/her health is concerned (Herbert *et al*., 2009).

The interplay between stress and self-empowerment has long concerned the scientific community. In summary, chronic or psychological stress has detrimental effects on daily decision-making and may lead to psychiatric disorders such as depression (Sousa *et al.*, 2000; Radley *et al.*, 2004; Cerqueira *et al.*, 2007; Pariante and Lightman, 2008).

Stress also has a detrimental effect on sleep. The increased emotional arousal is also involved as a main factor in the pathophysiology of chronic insomnia (Baglioni *et al.*, 2010). Chronic insomnia is associated with stress-related disorders, such as depression and anxiety (Akerstedt *et al.*, 2007; Li *et al.*, 2018).

Different strategies have been developed to patients’ active participation in decisions regarding their health (Colombo *et al.*, 2012). The spectrum varies from systems where patients lead the reform of health care services such as empowerment which is a critical tool for enhancing health protection and prevention in different intervention cases and diseases in contrast with a paternalistic approach that still limits patients’ autonomy in decision-making. Many studies indicate that empowerment is correlated with depression (Morin *et al.*, 2006; Bartlett *et al.*, 2008; Porter and Bejerholm, 2008), self-efficacy (Fuller *et al.*, 2015), and quantity and quality of sleep (Andrich, 1982; Mercer *et al.*, 2007). More precisely, patients with high levels of empowerment seemed to experience lower depression severity levels, higher self-efficacy, and better quality and quantity of sleep (Rush and Sharma, 2016).

Several clinical studies have evaluated the effect that there is also a strong connection between cancer and empowerment (Holland, 2002), as well as between cancer and chronic insomnia. In the current study, we found that the highest rates of chronic insomnia were found in breast cancer patients (Savard *et al.*, 2009). In a recent study, the Distress – Empowered Management Through Mind-Body Therapies in Oncology such as mediation, yoga, hypnosis, relaxation, and imagery, around cancer diagnosis have shown a reduction in psychological symptoms including depression, anxiety, insomnia, and fatigue (Carlson, 2017). The field today addresses the emotional and psychological responses of patients that impact on cancer morbidity and mortality (Carlson *et al.*, 2012). We chose the Patient Empowerment Strategies Questionnaire (PES-Q) because of its correlation with various types of cancer, secondly, due to its wide range of its questions,and, thirdly, in order to examine how this scale affects insomnia levels individually by measuring the patients’ active role in managing their own health.

The PES-Q consists of general statements covered to relevant and timely information about their illness, social support networks, open and positive communication between health professionals themselves and with the patient, decision-making involvement, and adjustment to acceptance of their illness. Other topics of importance included use of complementary therapies, religion and/or spirituality, and feeling useful in terms of paid/unpaid employment.

There is a little reference of empowerment scales related to the Greek population. We found a scale measuring the empowerment in Greek population through daily activities, but no one specified in chronic insomnia (Darviri *et al.*, 2014).

The rationale behind choosing patients with chronic insomnia is the fact that is associated with many health and psychological-related negative repercussions. The rationale behind selecting this particular questionnaire lies in that it is easy to administer, fast and comprehensive and that it can lead to specific results that can be used by a therapist. This study aimed to validate the PES-Q (Bulsara and Styles, 2013) in Greek patients suffering from chronic insomnia, in order to indicate the importance of health promotion and to prove whether an empowered individual, capable of controlling stress and making decisions, has a long-term benefit in their sleep quality (Starcke and Brand, 2012). The notion of helping patients with chronic insomnia through empowerment is innovative and promising (Bower and Irwin 2016). This fact can give an extra value to our study.

## Materials and methods

### Procedure

Translation was carried out using forward–backward translation method by two independent researchers. The Greek version was pretested on a small sample of five individuals, in order to trace unclear parts of the questions and to determine the final assessment of the translation. All rights of the PES-Q are reserved by its authors, thus we acted accordingly, starting the validation study after having received the appropriate license from the creators.

We distributed the questionnaires between April 2016 and July 2017. The sample was recruited from the Attikon General Hospital, School of Medicine, University of Athens. The diagnoses were established through clinical evaluation, observations, and diagnostic interview by a neurologist, who was specialized in the field of sleep disorders, according to guidelines of standard criteria (International Classification of Sleep Disorders-Third Edition (ICSD-3); American Psychiatric Association, 2013). The participants were chronic insomnia outpatients between 18 and 85 years old, who had the ability to read and write in Greek. The study received ethical approval from the Hospital Ethics Committee as it was found consistent with the Declaration of Helsinki (Washington, 2013). All patients gave their informed consent. We thoroughly informed the participants for the purpose of the research, and they voluntarily offered to complete the questionnaires. Data were collected in an anonymous and confidential manner as participants did not record their names during completion of the test and the researchers analyzing the data were not capable to attribute any of the tests to a specific participant. This implies that completion was honest, objective, and spontaneous. During the questionnaires’ completion, no questions arose due to lack of clarity. All participants were advised to leave blank any questions which they did not wish to answer. The average completion time was 30 min. The tool was easy to understand and patient compliant, and compared to other studies (Buysse *et al.*, 1989; Johns, 1991), the length of time for completion of questionnaire required about 5 min to be completed. The return rate of the distributed questionnaires was 90%. From this process, our sample number reached 110, of which 17 did not complete the entire questionnaire or did it in a random way. Our final sample consisted of 93 patients.

### Sample

The validation was carried out in a sample of 93 adult patients with chronic insomnia. From the 93 chronic insomniacs, 26 were males and 67 were females. Of these, 35 subjects were married, 46 divorced, 7 widowed, and 15 single. Their mean age was 54.7 years (SD = 15.2; range: 18–85 years). About 33% of the sample ranged from 39.5 to 54.6 years, 33% of the sample ranged from 54.7 to 69.9 years, 13% of the sample was from 24.3 to 39.4 years and 13% of the sample ranged from 70 to 82.4 years. The sample size was determined after careful examination of time restrictions and patient availability. The sample size was in accordance with several contemporary studies where clinical population is measured (*N* < 100) (Hill, 1998). The survey was conducted in the outpatient ‘sleep disorders’” clinic of the Neurology Department of Attikon University Hospital, National and Kapodistrian University of Athens, Greece. All participants in the sample were administered in the neurology ward of the hospital. Most of them were diagnosed for chronic insomnia or relative psychological symptomatology such as stress, depression and general agitation and psychological discomfort. All participants were of Greek nationality meaning that they had been born in Greece or were given the right to stay permanently in the country according to law standards. The participation to the study was voluntary and all participants signed a written consent form prior to test battery completion. About 90% of the participants completed the test. The remaining 10% who did not complete the test did not give specific reasons for not doing so. The 10% were mostly questionnaires not thoroughly completed with many missing values. The participants completed the tests in the hospital at the neurology ward.

### Tools

#### PES-Q created by Bulsara and Styles

The PES-Q is a self-administered tool originally created for English-speaking populations. Main themes initially identified were patient control of illness, patient choice of treatment location (in relation to the Shared Care model), patient information, support from others (non-medical), feeling useful, spirituality, use of complementary therapies, and acceptance of the illness. It consists of 15 questions that are rated on a Likert scale from 1 (‘I totally agree’) to 4 (‘I totally disagree’). Higher scores are indicative of higher empowerment of the individual. The following scales were administered in order to assess the predictive validity of the PES-Q (Bulsara and Styles, 2013). Selecting for four options in a Likert Scale question is indicative of the authors’ desire to force participants to choose a specific answer in every item. This pattern is common in situations where indecisiveness may appear or other polosis hazards. Also in many other scales like the very popular Depression, Anxiety and Stress Scale (DASS-21), four options are used.

#### Depression Anxiety Stress Scale

The DASS-21 scale is a questionnaire with three subscales: depression, anxiety, and stress. Each subscale includes seven questions in which the person is asked to answer through a four-level Likert-type scale from 0 (ie, not applicable to me at all) to 3 (ie, it was too or more often for me). Higher scores indicate greater levels of depression, anxiety, and stress experienced by the individual. The DASS-21 is widely used for clinical and research purposes and has good psychometric properties (Lyrakos and Arvaniti, 2009; Lyrakos *et al.*, 2011).

### Self-Efficacy Scale

The Self-Efficacy Scale (SES) consists of ten self-efficacy questions, where the individual is asked to answer how he/she feels for him/herself. One can choose among the following answers that are rated on a Likert scale from 1 (No Truth), to 4 (Totally Truth). The SES is widely used for clinical and research purposes and has good psychometric properties (Schwarzer and Jerusalem, 1995).

#### Athens Insomnia Scale

The Athens Insomnia Scale (AIS) is an eight-item standardized self-assessment psychometric instrument designed for quantifying sleep difficulty based on the International Classification of Diseases, Tenth Revision (ICD-10) criteria. It consists of eight items: the first five pertain to sleep induction, awakenings during the night, final awakening, total sleep duration, and sleep quality while the last three refer to well-being, functioning capacity, and sleepiness during the day. Total AIS scores range from 0 to 24, with a higher score indicating greater insomnia symptom severity. The AIS is widely used for clinical and research purposes and has good psychometric properties (Soldatos *et al.*, 2003).

#### Pittsburg Sleep Quality Index

The Pittsburg Sleep Quality Index (PSQI) is used to assess quality of sleep. It consists of nine questions related to sleep habits during the last 30 days. The individuals are asked to answer questions based on a Likert-type scale (0 = not in the last 30 days, 1 = less than once a week, 2 = once or twice a week, 3 = three or more times a week; 0 = no problem, 1 = only one minor problem, 2 = quite a problem, 3 = very big problem; 0 = very good, 1 = rather good, 2 = rather bad, 3 = very bad). The PSQI is widely used for clinical and research purposes and has good psychometric properties (Kotronoulas *et al.*, 2010).

### Statistical analysis

In order to examine the psychometric properties of the PES-Q, statistical analysis was executed through the use of the IBM SPSS 23 software package (IBM SPSS Statistics, Armonk, NY, USA) and R (version 3.2.4; R Foundation for Statistical Computing, Vienna, Austria).

Descriptive statistics were applied for all variables in the study, namely, demographics, means, and SD of the PES-Q and the other tools. After that we conducted an interitem correlational analysis of the PES-Q in order to discover possible anomalies in the behavior of the items. Finally, reliability, factor, and validity (criterion) analyses were performed in order to empirically validate the questionnaire.

## Results

### Descriptive statistics

The mean score of PES-Q was *M* = 32.99 and the SD was 7.10. Scores range from 15 (minimum) to 54 (maximum). According to these results, scores lower than 19 are indicative of low empowerment, scores ranging from 26 to 40 are indicative of normal empowerment, and scores exceeding 47 are indicative of extremely high level of empowerment.

### Interitem correlations

Initially, we tested the correlations between all items of the questionnaire. High positive correlations may be indicative of overlapping of some items, while high negative correlations may suggest problematic items and content validity issues. Results showed that most interitem correlations were positive, ranging between *r* = 0.2 and 0.56, statistically significant at the *P* < 0.05 level.

### Reliability analysis

The reliability of the PES-Q was tested using the Cronbach’s *α*. Results showed a satisfactory level of internal reliability Cronbach’s *α* = 0.887. We also performed an item analysis examining the possibility of change of the internal reliability with the omission of specific items. The analysis showed that all items contributed equally to PES-Q’s reliability (range of Cronbach’s *α* = 0.873–0.888).

### Factor analysis

Exploratory principal component analysis was used to identify the factors from the PES-Q. Bartlett’s test was used to assess whether the correlation between the items was adequate; in contrast, a determinant value was calculated to assess the unwanted overcorrelation of items (determinant should be close to zero). The Kaiser–Meyer–Olkin statistic was used to assess the sample adequacy. The appropriate numbers of derived factors were identified initially due to the scree plot.

Items with loadings above 0.3 were examined as candidate components of the corresponding factor. Cronbach’s *α* values were calculated to assess the internal consistency. The scores were calculated and assessed for meaningful associations with other measurements of the study using Pearson’s *ρ* correlation coefficient. The level of significance *P* was 0.05.

**Figure 1.**
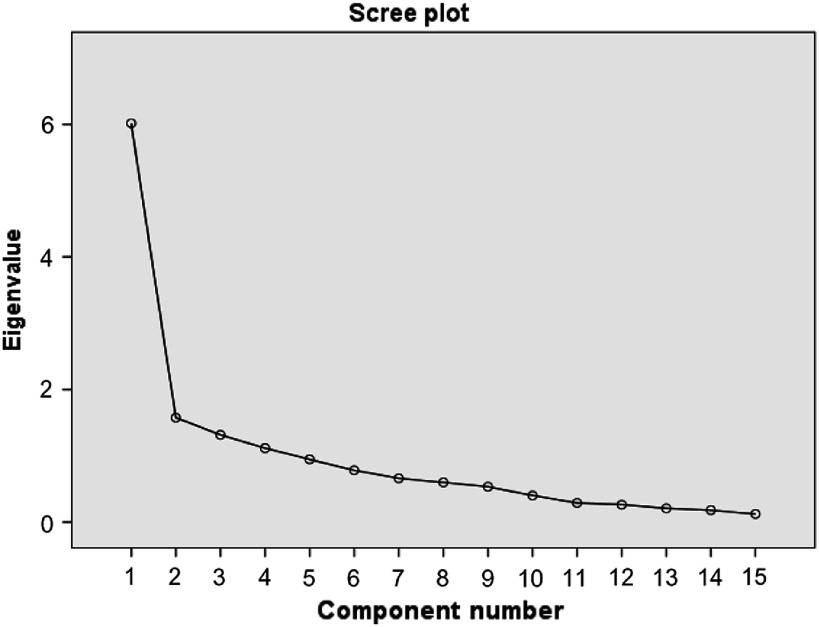
The scree plot of the PES-Q factors

The Kaiser–Meyer–Olkin (Table 1) measure was well above the 0.5 cutoff (ie, 0.789), confirming the sample adequacy. Determinant was close to zero attesting the absence of overcorrelation among items. The Barlett’s test of sphericity was significant [*F*(105) = 699.922, *P* < 0.0001], indicating satisfactory correlations among items. Based on the eigenvalues, the four-factor solution explained 66.8% of total variance. However, due to the size of the study sample, the Kaiser criterion was not valid, thus inflexion points in the scree plot (Figure 1) were used to ascertain the number of factors. Inspection of the scree plot revealed the evidence of the one-factor solution being the best possible model (ie, one inflexion point).

**Table 1.** Item loadings on the one-factor solution of the principal component analysis

Items	Loading
1. I am capable of handling my illness.	0.731
2. I have all the information I need to manage my illness.	0.713
3. I am capable of helping health professionals reach decisions related to my illness.	0.467
4. My family is very supportive.	0.452
5. I need the support of my family and friends.	0.428
6. My family and friends still rely on me.	0.754
7. I can adapt to the changes in my lifestyle.	0.738
8. Health professionals are happy to include me in decisions related to my illness.	0.681
9. I want my family and friends to continue to rely on me.	0.456
10. My friends are always supportive.	0.565
11. I still feel useful in my daily life.	0.658
12. My spiritual beliefs help me cope with my illness.	0.772
13. I accept that I have to change my lifestyle.	0.525
14. Complementary therapies help me cope.	0.679
15. I have a lot of confidence in my local GP.	0.706
Eigenvalue	6.016
Variance explained	40.11%
Cronbach’s *α*	0.887

The one-factor solution explained 40.1% of the total variance. Table 1 presents the results of the factor analysis for one factor. Based on the factor loading cutoff of 0.3, all items were kept in the one-factor solution predictive validity

Empowerment was positively correlated to quality of sleep (*r* = 0.318, *P* < 0.01) and self-esteem (*r* = 0.425, *P* < 0.001) and negatively correlated with insomnia (*r* = −0.226, *P* = 0.029), stress (*r* = −0.455, *P* < 0.001), anxiety (*r* = −0.420, *P* < 0.001), and depression (*r* = −0.506, *P* < 0.001). There were no significant associations between PES-Q and age (*P* = 0.497) or gender (*P* = 0.916).

## Discussion

Empowerment impacts in a positive way how patients react to treatment and illness side effects (Aujoulat *et al.*, 2008). Patients with high levels of empowerment achieve a level of control through adapting lifestyles and taking advantage of a range of supports (Neckelmann *et al.*, 2007). Patient empowerment describes a situation in which individuals are encouraged to take an active role in managing their health and become more involved in decision-making about their life (Anderson and Funnell, 2005; Aujoulat *et al.*, 2007).

The concept of empowerment has a high correlation with stress symptomatology such as anxiety, depression, and chronic insomnia. Also chronic or psychological stress has detrimental effects on daily decision-making and this fact may lead to psychiatric disorders (Baglioni *et al.*, 2010). Although many studies indicate that empowerment is correlated with depression (Taylor *et al.*, 2007; Siriwardena *et al.*, 2010), there is a little reference regarding to chronic insomnia. Circadian rhythms are centrally (within the central nervous system) and peripherally (within cells) regulated and stress hormones mediate the overall control (Kino and Chrousos, 2011). Disruption of daily rhythms is a major stressor that affects physiology and, over time, leads to disease (Kino and Chrousos, 2011). A good surrogate marker for the integrity of circadian rhythms is sleep quality (Buckley and Schatzberg, 2005; Lightman, 2008; Kino and Chrousos, 2011). Conceptually, treatment preference for chronic insomnia represents the outcome of a dynamic decision-making process that comes via empowerment (Sidani *et al.*, 2009).

The objective of this study was to validate the PES-Q in Greek Adult Patients with Chronic Insomnia, as a useful and promising instrument for evaluation of chronic insomnia symptomatology. Moreover, we chose to adapt this scale in Greek patients with chronic insomnia since physiological hyperarousal of emotional dysregulation and cognitive process has been identified as a key factor in chronic insomnia (Gratz and Roemer, 2004; Palagini *et al.*, 2017). PES-Q has not been validated in more studies and it is the first time a study was carried out in the Greek population.

The present study provides preliminary support for the reliability and validity of a Greek version of thePES-Q. Adaptation was based on data collected from 93 individuals, residing in urban areas, using principal component analysis. The questions loading onto the one factor were well satisfactory. Analysis of the psychometric properties of the PES-Q has the potential to identify areas of strength and potential future utilization. Results showed that the Greek version of the PES-Q has satisfactory reliability and validity in chronic insomniacs.

The PES-Q is a useful general clinical tool, is easy to complete and very simple to score which make it a cost-efficient and time-saving instrument for evaluating empowerment as part of a psychopathology prevention strategy. It may be used in a wide range of diseases related to chronic stress, it is easy to understand, as evidenced by the content of the questions and by the extremely high response rate. Specifically, the participants reported to the investigator that the test size and questions were straightforward (an observational deduction). The investigator reported that no questions arose during completion of the questionnaire by the participants.

This study suffers from several limitations. First, it is not safe to yet generalize the results across the Greek population, as our study was a pilot validation on citizens of Athens and patients suffering from chronic insomnia. Different countries in the world, or even different regions within a country, are at different stages of the epidemiologic transition, exacerbated by a poor lifestyle, which includes a risk to developing psychosomatic diseases due to a stressful lifestyle (Steyn and McHiza, 2014). Second, a limitation might come from the fact that no test–retest reliability was performed. Third, a small sample size was used for data collection.

Future studies may investigate the behavior of this scale in other stress-related diseases. Also, they can focus on examining the psychometric qualities of the PES-Q Greek version in a larger representative sample from all over the region of Greece. It would be very interesting to evaluate if PES-Q is sensitive to change and determine cutoff score for empowerment. We could include Greeks from different regions of Greece, including a test–retest reliability testing, covering a greater range of professionals and locations and also evaluate if the PES-Q is a sensitive to change and determine cutoff score for empowerment. Nevertheless, the results of the current study allow us to recommend this tool for future Greek studies. This study provides substantial empirical support for the psychometric qualities of the PES-Q and can serve as a base to a larger sample or a main standardization study.
